# Supplementary material for: A molecular phylogeny of Geotrochus and Trochomorpha species (Gastropoda: Trochomorphidae) in Sabah, Malaysia reveals convergent evolution of shell morphology driven by environmental influences
Source: PeerJ. 2021 Feb 2;9:e10526. doi: 10.7717/peerj.10526 (PMC7863784; doi:10.7717/peerj.10526)
Supplement: File S1 — The data was extracted from GBIF on September 1, 2020, and the distribution map was generated by using the records with coordinates information. [file peerj-09-10526-s001.docx]

**Additional File 1.** The maps of distribution for (A) *Geotrochus* and (B) *Trochomorpha* species based on the records with coordinate information obtained from Global Biodiversity Information Facility (GBIF).

**
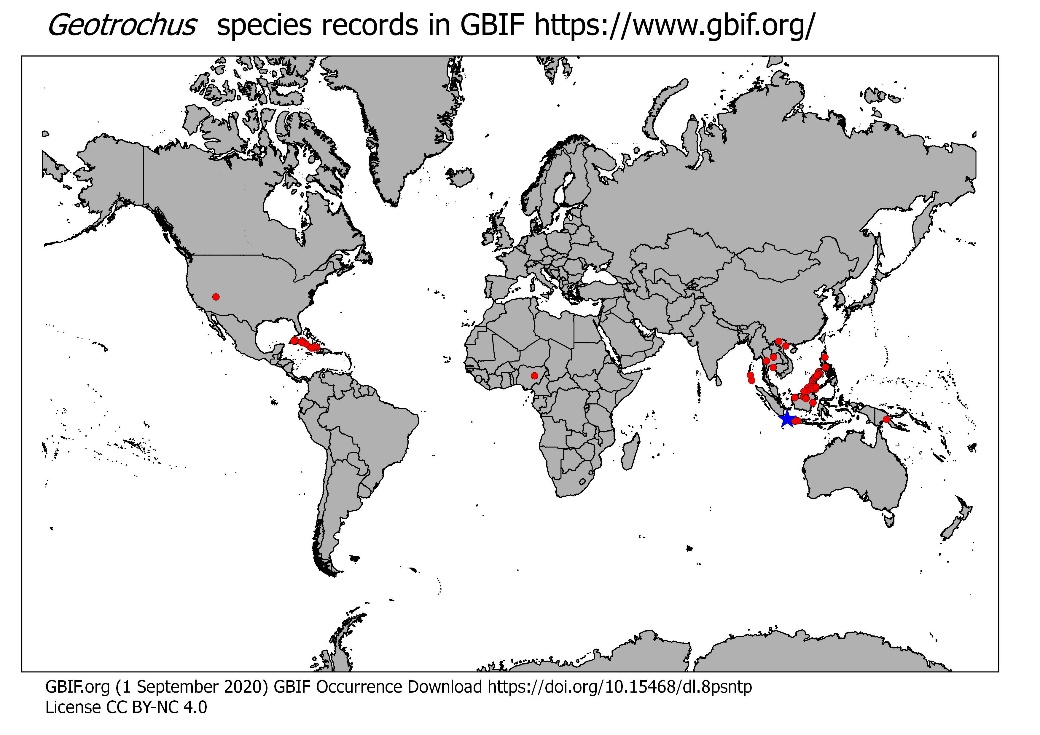
**

**Figure 1.**  *Geotrochus* species distribution map based on records in GBIF (red circles) and the type locality (blue star). The base map is from Global Administrative Areas ( 2012). GADM database of Global Administrative Areas, version 2.0. [online] URL: [www.gadm.org](http://www.gadm.org).

**
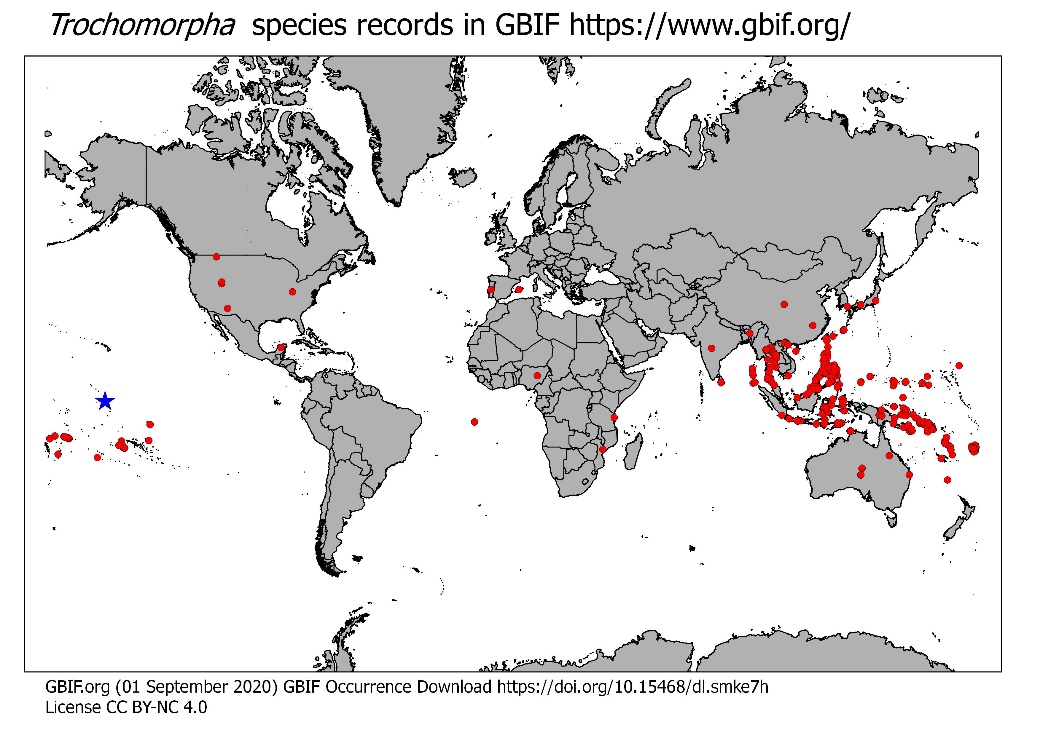
**

**Figure 2.**  *Trochomorpha* species distribution map based on records in GBIF (red circles) and the type locality (blue star). The base map is from Global Administrative Areas ( 2012). GADM database of Global Administrative Areas, version 2.0. [online] URL: www.gadm.org.
