# Supplementary material for: A molecular phylogeny of Geotrochus and Trochomorpha species (Gastropoda: Trochomorphidae) in Sabah, Malaysia reveals convergent evolution of shell morphology driven by environmental influences
Source: PeerJ. 2021 Feb 2;9:e10526. doi: 10.7717/peerj.10526 (PMC7863784; doi:10.7717/peerj.10526)
Supplement: File S2 — Each of the five partitions, namely, codons of COI, namely, 1st, 2nd and 3rd codon positions of COI, 16S rDNA, and ITS-1, was tested for molecular evolution via ModelFinder (Kalyaanamoorthy et al., 2017) and partition models (Chernomor, Von Haeseler & Minh, 2016) based on the both AIC and BIC that built into IQ-Tree v.1.6.7 (Nguyen et al., 2015; Trifinopoulos et al., 2016). We limited the candidate models to the six models that are available in MrBayes analysis, namely, JC, F81, K80, HKY, SYM and GTR. [file peerj-09-10526-s002.docx]

**Additional File 2.** Results of ModelTest for each partition of DNA sequencing alignment (16S, COI, and ITS), and the best partition schemes and substitution models for concatenated sequence data matrix. Each of the five partitions, namely, codons of COI, namely, the 1st, the 2nd and the 3rd codon positions of COI, 16S rDNA, and ITS-1, was tested for molecular evolution via ModelFinder (Kalyaanamoorthy et al., 2017) and partition models (Chernomor, Von Haeseler & Minh, 2016) based on the both AIC and BIC that built into IQ-Tree v.1.6.7 (Nguyen et al., 2015; Trifinopoulos et al., 2016). We limited the candidate models to the six models that are available in MrBayes analysis, namely, JC, F81, K80, HKY, SYM and GTR.

**Table 1.** Model of evolution and best partition scheme for each of the three genes for respective phylogenetic analysis.

| **Genes** | **Maximum Likelihood** | **Bayesian Inference** |
| --- | --- | --- |
| **16S** | K3Pu+F+G4 | GTR+F+G4 |
|  |  |  |
| **COI** |  |  |
| COI 1^st^ codon | K3Pu+F+R2 | GTR+F+G4 |
| COI 2^nd^ codon | TN+F+I | HKY+F+I |
| COI 3^rd^ codon |  |  |
|  |  |  |
| **ITS-1** | K2P+G4 | K80+G4 |

**Table 2.** Model of evolution and best partition scheme for concatenated DNA data matrix of the three genes for respective phylogenetic analysis.

| **Genes** | **Length of alignment** | **Maximum Likelihood** | **Bayesian Inference** |
| --- | --- | --- | --- |
| 16S | 461 | HKY+F+I+G4 | HKY+F+I+G4 |
| COI 1^st^ codon | 218 |  |  |
| COI 2^nd^ codon | 217 | TNe+G4 | K80+I |
| COI 3^rd^ codon | 217 |  |  |
| ITS-1 | 805 |  |  |
