# Supplementary material for: A molecular phylogeny of Geotrochus and Trochomorpha species (Gastropoda: Trochomorphidae) in Sabah, Malaysia reveals convergent evolution of shell morphology driven by environmental influences
Source: PeerJ. 2021 Feb 2;9:e10526. doi: 10.7717/peerj.10526 (PMC7863784; doi:10.7717/peerj.10526)

**Additional File 7.** Bayesian inference trees of Geotrochus and Trochomorpha species based on each of the (A) 16S, (B) COI and (C) ITS genetic markers. Support values on branches are Bayesian posterior probability (BI) followed by maximum likelihood (ML) bootstrap value. The number shown beside each specimen is same as the specimen number in Table 2, and the specimens with red curly bracket and red arrows are taxa that incongruent with the clades in the phylogenetic tree estimated based on the concatenated sequences data matrix (Figure 3).


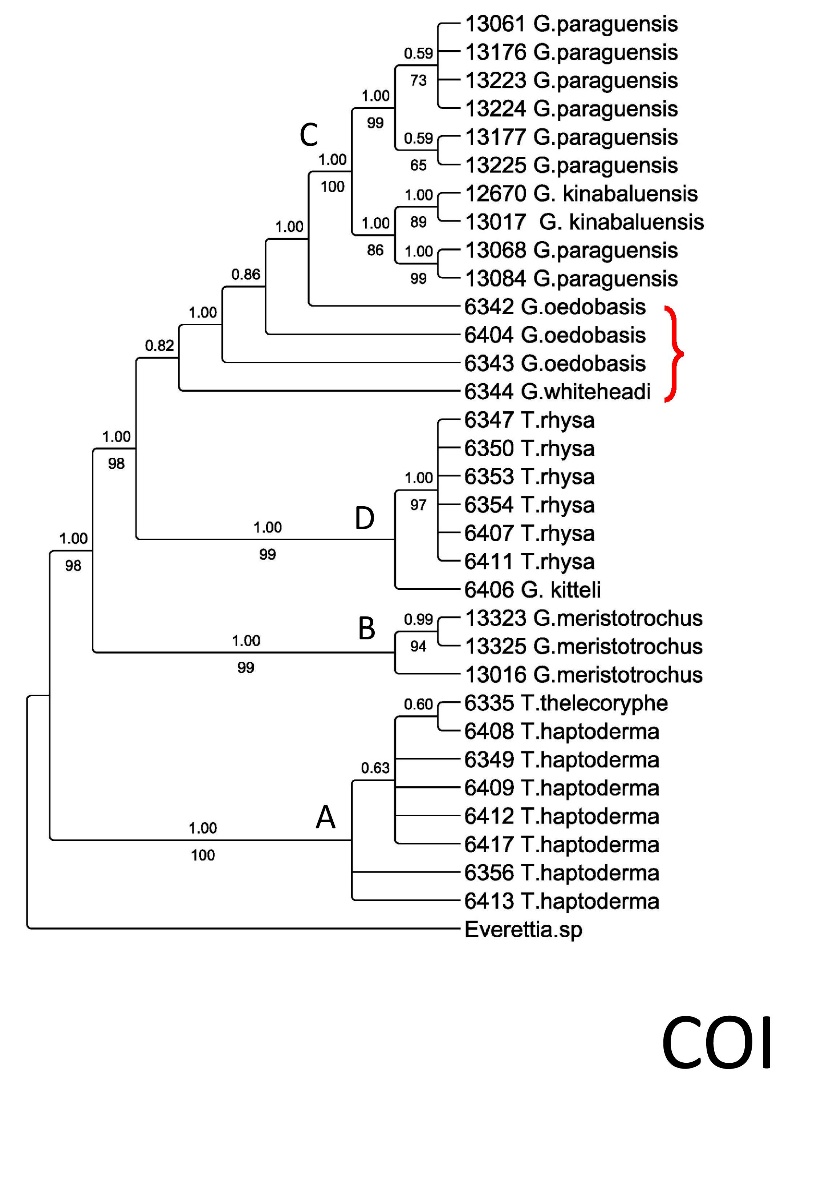


**B**


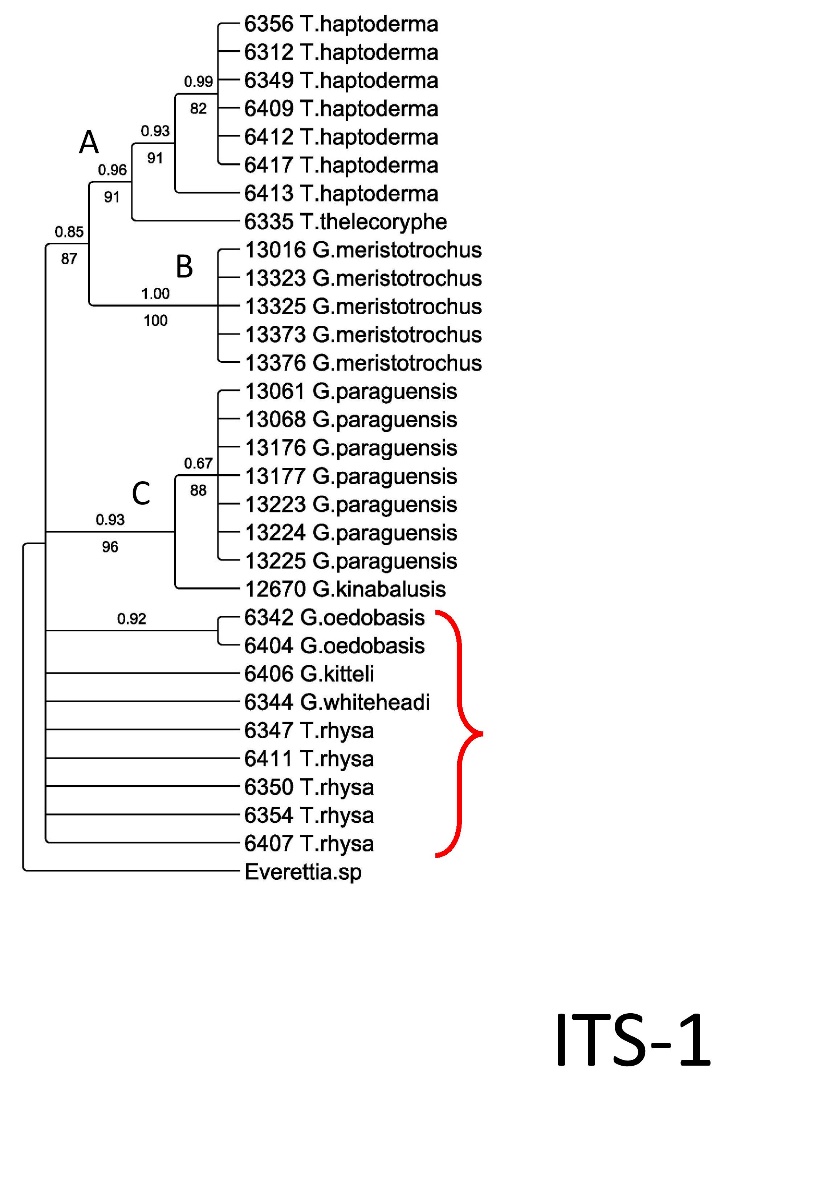


**C**

**A**


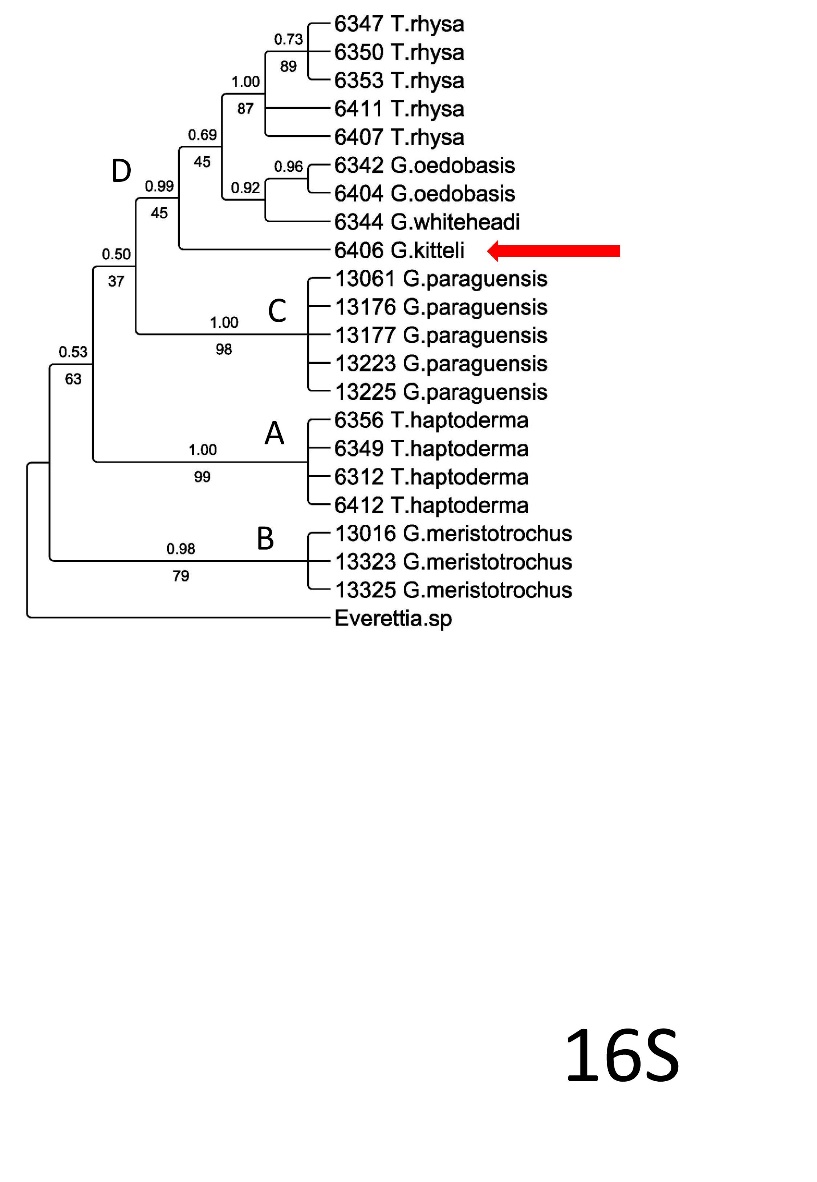

Supplement: File S7 — Support values on branches are Bayesian posterior probability (BI) followed by maximum likelihood (ML) bootstrap value. The number shown beside each specimen is same as the specimen number in Table 3, and the specimens with red curly bracket and red arrows are taxa that are incongruent with the clades in the phylogenetic tree estimated based on the concatenated sequences data matrix. [file peerj-09-10526-s007.docx]
